# *Brucella* deploys multiple type IV effectors that simultaneously control bacterial survival and host inflammatory cell death

**DOI:** 10.1080/21505594.2026.2721826

**Published:** 2026-08-28

**Authors:** Dongjie Sun, Mengtao Zhang, Yifan Wu, Enhui Dai, Xuezheng Fan, Yanxiao Zhao, Jiabo Ding

**Affiliations:** a Key Laboratory of Animal Biosafety Risk Prevention and Control (North) of MARA, Institute of Animal Science, Chinese Academy of Agricultural Sciences, Beijing, China; b College of Animal Science and Veterinary Medicine, Shenyang Agricultural University, Shenyang, China

**Keywords:** *Brucella*, novel T4SS effector proteins, bacterial morphology, pathogenicity, cell death

## Abstract

The type IV secretion system (T4SS) acts as the central virulence determinant of *Brucella*, facilitating intracellular survival via the secretion of effector proteins. In this study, we combined bioinformatic prediction with translocation assays to identify seven novel VirB-dependent effectors. Functional characterization revealed distinct roles for these proteins in both bacterial physiology and host-pathogen interactions. We identified BT4E19 and BT4E43 as critical determinants of cell envelope integrity: BT4E19 is required for core oligosaccharide maintenance and nitrosative stress tolerance, whereas BT4E43 is essential for O-antigen biosynthesis and oxidative stress resistance. Furthermore, BT4E43 is required for the efficient avoidance of lysosomal trafficking during intracellular infection. Notably, BT4E4 displays dual functions under tested conditions, being essential for oxidative stress resistance while simultaneously functioning to inhibit Caspase-5-mediated pyroptosis. *In vivo* assays further demonstrated that both BT4E19 and BT4E43 are indispensable for establishing chronic infection in mice. Collectively, these findings expand the *Brucella* effector repertoire and uncover the dual functions of specific effectors in maintaining bacterial structural integrity and orchestrating immune evasion, highlighting them as potential targets for anti-virulence therapies.

## Introduction

*Brucellosis*, caused by facultative intracellular bacteria of the genus *Brucella*, remains a significant zoonotic disease worldwide, affecting a broad range of mammalian hosts, including humans and livestock [1,2]. A critical determinant of *Brucella* pathogenicity is its ability to survive and replicate within host macrophages, a process dependent on the formation of a specialized intracellular niche known as the *Brucella*-containing vacuole (BCV) [3,4]. The maturation of the BCV and the subsequent intracellular proliferation of the bacterium are strictly governed by the VirB type IV secretion system (T4SS), a multiprotein complex encoded by the virB operon [3,4]. The T4SS functions by translocating bacterial effector proteins into the host cell, where they modulate diverse host cellular processes to favor bacterial persistence and immune evasion [5–7].

Despite the established centrality of the T4SS in *Brucella* virulence, the complete repertoire of its translocated effectors remains incompletely defined. While several effectors have been characterized, the complexity of host-pathogen interactions suggests that many substrates responsible for subverting host functions are yet to be discovered [8,9]. Identification of these effectors is essential for elucidating the molecular mechanisms underlying *Brucella* intracellular survival and chronic infection. Recent advances in bioinformatics, such as the S4TE 2.0 prediction algorithm, coupled with experimental translocation assays like the adenylate cyclase (CyaA) and β-lactamase (TEM1) reporter systems, offer powerful integrated approaches for the systematic discovery of novel T4SS substrates [10,11].

Beyond their roles in manipulating host signaling, T4SS effectors may also contribute to bacterial physiological fitness. The structural integrity of the bacterial envelope, particularly the lipopolysaccharide (LPS), is vital for resistance to harsh intracellular environments and evasion of host immune recognition [12]. Defects in LPS biosynthesis, such as the loss of O-antigen or alterations in the core oligosaccharide, typically result in attenuation of virulence and increased susceptibility to host defenses [13,14]. There is already evidence indicating that some *Brucella* T4SS effectors may possess dual regulatory functions. For example, the recently identified T4SS effector RS15060 was shown to concurrently modulate bacterial morphology, lipopolysaccharide (LPS) core synthesis, and host proinflammatory responses, thereby promoting *Brucella* virulence [15]. However, the intersection between T4SS effector function and the maintenance of bacterial cell envelope integrity remains an underexplored area of *Brucella* biology.

Furthermore, the interplay between *Brucella* infection and host cell death pathways is a critical aspect of pathogenesis. Pyroptosis, a lytic form of programmed cell death driven by inflammatory Caspases and accompanied by the release of pro-inflammatory cytokines like interleukin-1β(IL-1β), serves as a key host defense mechanism [16,17]. *Brucella* has evolved strategies to modulate these inflammatory responses to establish a stealthy infection [18,19]. Understanding how specific T4SS effectors modulate pyroptosis and other inflammatory pathways is crucial for deciphering the immune evasion tactics of this pathogen.

In this study, we integrated computational prediction with experimental validation to identify seven previously uncharacterized VirB-dependent effectors. BT4E43 is essential for O-antigen biosynthesis and oxidative stress resistance and is required for avoidance of lysosomal fusion and is also required for chronic infection in mice. BT4E19 is required for core oligosaccharide maintenance and required for chronic infection in mice. Furthermore, we demonstrate that while BT4E4 is essential for bacterial resistance to oxidative stress, it simultaneously functions as a direct inhibitor of Caspase-5–mediated pyroptosis. These findings, confirmed by the indispensability of these effectors for chronic infection in mice, expand the *Brucella* effector repertoire and highlight a sophisticated strategy where effectors serve as dual regulators of bacterial physiology and host immune subversion.

## Materials and methods

### Reagents

For immunofluorescence staining or Western analysis, the following antibodies were used: Rabbit anti-DYKDDDDK (Proteintech, #66008–4-Ig); Goat Anti-Mouse IgG (H+L), HRP Conjugate (TransGen, #HS201); Goat Anti-Rabbit IgG H&L (Alexa Fluor® 647) (Abcam, #ab150083). Intracellular cAMP levels were measured with the Cyclic AMP Direct ELISA Kit (ARBOR ASSAYS, #K019-H1), while the beta-lactamase activity was determined using the LiveBLAzer™ FRET-B/G Loading Kit with CCF4-AM (Thermo Fisher, #K1095).

### Bacterial strains and culture

The wild-type *Brucella abortus* reference strain 2308 was obtained from the China Institute of Veterinary Drug Control (IVDC). All *Brucella* strains were cultured in Tryptic Soy Broth (TSB) or on Tryptic Soy Agar (TSA) (BD Difco, Franklin Lakes, New Jersey, USA) at 37°C under 5% CO_2_. All manipulations involving live *Brucella* were performed in a Biosafety Level 3 (BSL-3) laboratory in compliance with relevant biosafety regulations.

### Plasmids

Vectors pBBR1MCS-1, pBluescript II KS (+), and pUC19-SacB were maintained in our laboratory. Screening vectors (D-pZL1790-TEM1/CyaA-2FLAG), suicide plasmids for gene deletion (containing ~1 kb homology arms), and complementation constructs (pBBR1MCS-1 backbone with native promoters) were generated via Gibson Assembly. Eukaryotic expression plasmids were constructed by cloning effector ORFs into p3xFLAG-CMV-7.1, pEGFP-N1, or pK5-MYC. All constructs were confirmed by DNA sequencing (Beijing Ribio Biotech), and primer sequences are listed in Supplemental Table S1.

### Brucella in-frame deletion mutants and genetic complementation

Suicide vector was introduced into *B. abortus 2308* by electroporation. Single-crossover integrants were selected on ampicillin plates, and double-crossover deletion mutants were obtained by sucrose counter-selection (5% sucrose) to promote the second recombination and plasmid loss. Deletions were confirmed by colony PCR and Sanger sequencing. For complementation, the resulting plasmid was electroporated into the corresponding mutant and complemented strains were selected on chloramphenicol; successful complementation was confirmed by Western blot analysis of target protein expression.

### Mammalian cell culture and bacterial infections

HeLa and 293T cells were cultured in high-glucose DMEM with 10% FBS, while Expi293F cells were maintained in FreeStyle™ 293 Expression Medium (Invitrogen, # 12338018) with shaking (130–150 rpm). Macrophage lines RAW264.7 and THP-1 were cultured in DMEM (Invitrogen, #11965092) and RPMI-1640 (Invitrogen, #11875093), respectively, supplemented with 10% heat-inactivated FBS (56°C, 30 min). THP-1 cells were differentiated with 100 ng/mL PMA for 48 h prior to use. All cells were obtained from ATCC and incubated at 37°C with 5% CO_2_.

For infection assays, RAW264.7/THP-1 (2.5 × 10^5^cells/well) and HeLa (2 × 10^4^cells/well) were seeded in 24-well plates and infected with *Brucella* strains at multiplicities of infection (MOI) of 100:1 and 1000:1, respectively. After 1 h of incubation, extracellular bacteria were eliminated by washing and treatment with 100 μg/mL gentamicin (Solarbio, # 1405–41-0) for 1 h. Cells were then maintained in medium containing 10 μg/mL gentamicin. At 1, 24, and 48 h post-infection (p.i.), cells were washed with PBS and lysed with 0.1% Triton X-100 to quantify intracellular colony-forming units (CFU) on TSA plates. All assays were performed in triplicate.

### Bacterial adherence and invasion

Prior to infection, the bacterial suspensions were serially diluted and plated to determine the exact input CFU. Cells were plated in the 24-well plates and infected with *Brucella* strains at an MOI of 100 CFU per cell. To synchronize the infection, the infected plates were centrifuged at 400 × g for 5 min, and cells were then incubated at 37°C with 5% CO_2_ for 1 h. Cells were washed twice with PBS to remove nonadherent bacteria. For adherence assays, wells of infected cells were incubated with 500 µL of 0.1% (v/v) Triton X-100 in sterile water for 15 min at 37°C and the solution of lysed cells was serially diluted 10-fold and the CFU of bacteria was determined by plating on TSA for 3–5 days at 37°C.

For invasion assays, the infected cells were washed twice with PBS and cultured in a medium containing gentamicin (100 µg/mL) to remove extracellular bacteria, and incubated at 37°C with 5% CO_2_ for 1 h. Cells were washed twice with PBS to remove gentamicin. CFU were counted to the invading bacteria.

### TEM1 assay

Plasmids with C-terminal fusions of the putative effectors to TEM1 (Supplemental Table S1) were electroporated into wild-type *Brucella abortus* 2308 or its isogenic Δ*VirB9* mutant. Positive transformants were cultured in tryptic soy broth (TSB) supplemented with kanamycin (50 μg/mL) (Solarbio, # 25,389–94-0) to an OD_600_ of 0.6–0.8 and induced with 1 mM IPTG (Solarbio, # 1405–41-0) for 2 h. The induced bacteria were used to infect RAW264.7 macrophages seeded in black-walled, clear-bottom 96-well plates at a density of 1 × 10^5^ cells per well (MOI = 1000), with 0.1 mM IPTG included in the infection medium to sustain induction. This MOI was necessary to achieve high levels of infection within the population and within individual cells and detect TEM fusion translocation [8]. At 1 h p.i. gentamicin (50 μg/mL) was added for 1 h to eliminate extracellular bacteria, after which plates were incubated further until 16 h p.i. Cells were washed twice with phenol red-free HBSS (Beyotime, # H1045) and loaded with CCF4-AM substrate prepared according to the manufacturer’s instructions (LiveBLAzer™ FRET-B kit, # K1095) in the presence of 5 mM probenecid. Following a 90-min incubation in the dark at room temperature, fluorescence was visualized using an inverted microscope equipped with a β-lactamase-specific filter set (excitation: 409 nm; emission: blue at 447 nm, green at 520 nm) [20,21]. For each well, at least 300 cells were analyzed across ten independent random fields to determine the percentage of blue fluorescent (TEM-1-positive) cells. Results are expressed as the mean ± standard deviation (SD) from three independent experiments.

### CyaA assay

CyaA adenylate cyclase protein translocation assays were performed essentially as previously described [8,22] with the following modifications. Plasmids with C-terminal fusions of the putative effectors to CyaA (Supplemental Table S1) were electroporated into wild-type *B. abortus* 2308 and its isogenic Δ*Virb9* mutant. Positive transformants were grown in TSB supplemented with kanamycin (50 μg/mL) and induced with 1 mM IPTG for 2 h prior to infection. RAW264.7 cells (2.5 × 10^5^ cells/well) seeded in 24-well plates were infected at an MOI of 1000 for wild-type strains or 5000 for Δ*Virb9* strains. These MOIs were necessary to achieve high levels of infection that allowed detection of cAMP production [8]. The infection procedure (centrifugation for adsorption, washing, and gentamicin treatment) was identical to that used for the TEM1 assay. At 6 h p.i., cells were collected cAMP concentrations were measured using the Cyclic AMP Direct ELISA Kit (Arbor assays, # K019-H1) strictly according to the manufacturer’s instructions. Absorbance was read at 450 nm and cAMP values were calculated using the online tool (www.myassays.com/arbor-assays-cyclic-amp-direct-eia-kit-non-acetyl.assay). Each sample was assayed in duplicate, and experiments were independently repeated three times.

### Scanning electron microscopy

For scanning electron microscopy (SEM), *B. abortus* 2308 and its derivative strains were fixed in 2.5% glutaraldehyde at 4°C for 24 h to inactivate bacteria while preserving cellular ultrastructure. To confirm complete inactivation, 100 μL fixed bacterial suspension was spread onto TSA agar plates. No colonies were observed after aerobic incubation at 37°C for 72 h. Fixed bacterial pellets were washed three times with pre-cooled PBS, followed by serial dehydration in 30%, 50%, 70%, 80%, 90%, 95%, and 100% ethanol. Samples were processed by critical point drying, mounted on conductive stubs, and sputter-coated with platinum prior to SEM observation.

Quantitative morphological analysis of *Brucella* was performed using ImageJ software. The length of individual bacterial cells was directly measured from SEM micrographs. The degree of cell swelling was determined by comparing bacterial width with that of wild-type *B. abortus* 2308. Structural damage, including surface distortion, membrane blebbing and membrane rupture, was assessed according to morphological characteristics. All measurements were performed in a blinded manner to minimize observer bias.

### Immunofluorescence microscopy

Cells were fixed with 4% paraformaldehyde (Solarbio,#P1110) for 10 min at room temperature. Cells were permeabilized and blocked in PBS containing 5% BSA and 0.1% Triton X-100 for 10 min, incubated with primary antibody (1:1000 in 5% BSA) for 1 h at room temperature, washed, and incubated with CoraLite488-conjugated goat anti-mouse secondary antibody (1:1000) (Proteintech,#SA00013-1) for 1 h in the dark. Nuclei were stained with DAPI (0.5 μg/mL) (Yeasen, # 40728ES03) for 10 min, and coverslips were mounted with anti-fade mounting medium. Images were acquired on a Leica TCS SP8 confocal microscope and processed with ImageJ.

### Stress assays

Stress tolerance was evaluated following established protocols [23] with modifications. Bacterial suspensions (1 × 10^6^ CFU/mL) were exposed to SDS (100 µg/mL) (Beyotime, # S8010), ethanol (10%) (Merck, #459828), or polymyxin B (500 µg/mL) (Beyotime, # P8350) for 2 h, or to acid peptone water (1 g/L Tryptone, 5 g/L NaCl, pH = 6.5, 5.5, or 4.5) [23]for 2 h with shaking. Long-term survival under osmotic, iron-limiting, or heat stress was assessed by culturing bacteria in TSB containing 400 mM NaCl or 10 mM 2,2’-dipyridyl (DIP) (Merck, #798533), or at 42°C, with CFU quantification at 24 and 48 h. Survival was determined by spot-plating serial dilutions and expressed relative to controls. All experiments were performed in biological triplicates.

### Lipopolysaccharide (LPS) extraction, crystal violet staining, acriflavine agglutination test, and silver staining

LPS was extracted using a commercial kit (iNtRON, #17141). O-antigen integrity was evaluated via crystal violet staining and acriflavine agglutination as previously described [23,24]. Briefly, colonies were stained with 0.05% crystal violet for 30 s. For agglutination, bacterial suspensions were mixed with equal volumes of 0.05% acriflavine (Sigma-Aldrich,#A8126) and incubated at 80°C for 2 h. To validate the specificity of the reaction, two additional controls were included: a “bacteria only” control, in which the bacterial suspension was mixed with sterile PBS instead of acriflavine to assess baseline auto-agglutination or turbidity; and an “acridine yellow” control, in which the acridine yellow reagent alone was subjected to heat treatment, to rule out agglutination-like artifacts caused solely by heat-induced physical changes. Extracted LPS was resolved by 12.5% SDS-PAGE and visualized by silver staining (FITGENE, #FI8921). In this assay, the WT strain and R6/66 were used as positive and negative controls, respectively.

### Quantitative real-time polymerase chain reaction (qRT-PCR)

Total RNA was extracted with the RNA Easy Fast kit (Tiangen, #DP451). One microgram of RNA was reverse transcribed with HiScript II Q Select RT SuperMix (+gDNA wiper) (Vazyme, #R223-01). qPCR was performed using Taq Pro Universal SYBR qPCR Master Mix (Vazyme, # Q712-02) on a StepOnePlus system. Reaction conditions were 20 μL total volume, 95°C for 1 min, followed by 40 cycles of 95°C for 10 s and 60°C for 30 s. Target genes included *Caspase1*, *Caspase4*, *Caspase5,* and *GSDMD*; *GAPDH* or β-actin was used as reference. Relative expression was calculated by the 2^−ΔΔCt^ method. Primer sequences are listed in Supplemental Table S1.

### Flow cytometry

Expi293F suspension cells transfected with p3xFLAG-CMV-7.1-BT4E or empty vector were harvested 30–36 h post-transfection. Approximately 1 × 10^5^ cells per sample were collected, stained with Annexin V-FITC/PI (Beyotime, # C1062S) according to the kit instructions and analyzed by flow cytometry. Data were processed with Flow Jo. Each condition was run in triplicate and experiments were independently repeated three times.

### Mice infection

Female BALB/c mice (4–6 weeks old, SPF grade) were acclimated for one week before use and randomly assigned to groups. Groups of five mice were infected intraperitoneally (i.p.) with 10^5^ CFU of the indicated *Brucella* strains. At 1-, 2-, 4-, and 6-weeks p.i. mice were anesthetized with sodium pentobarbital (50 mg/kg, i.p.) and euthanized by cervical dislocation. Spleens were aseptically collected, weighed, and homogenized in 3 mL PBS. Serial dilutions of the homogenates were plated on TSA for CFU enumeration. Data are presented as the mean ± standard deviation (SD) of log-transformed CFU/spleen(*N* = 5).

### Ethics statement

Female BALB/c mice (4–6 weeks old, SPF grade) were purchased from Beijing Vital River Laboratory Animal Technology Co., Ltd. (Beijing, China) and acclimated for one week before use. Animal experiments were carried out in strict accordance with the Guide for the Care and Use of Laboratory Animals and were approved by the Institutional Animal Care and Use Committee at Institute of Animal Science, Chinese Academy of Agricultural Sciences (Permission: IAS2025-38). All experiments with *Brucella* in the present study were performed within the biosafety level 3 (BSL-3) facilities in the Lanzhou Veterinary Research Institute, Chinese Academy of Agricultural Sciences approved by the Ministry of Agriculture and Rural Affairs and China National Accreditation Service for Conformity Assessment.

This study was conducted in accordance with the ARRIVE (Animal Research: Reporting of In Vivo Experiments) guidelines to ensure transparency, reproducibility, and ethical rigor in the reporting of animal research.

### Statistical analysis

All experiments were performed with at least three independent biological replicates. For pairwise comparisons, two-tailed unpaired Student’s *t*-tests were applied. Multi-group analyses utilized one-way ANOVA with Tukey’s multiple comparisons test. Data are presented as mean ± standard deviation (SD). Statistical analyses and graphing were carried out using GraphPad Prism 9.0. All tests *p* value < 0.05 were considered statistically significant. Significance in figures is indicated as: ns, not significant; *p < 0.05; **p < 0.01; ***p < 0.001; ****p < 0.0001.

## Results

### Prediction of putative Brucella T4SS effector proteins

To screen the putative *Brucella* T4SS effector proteins, we systematically analyzed the *Brucella* genome using the S4TE 2.0 database (Searching Algorithm for Type IV Effector proteins 2.0, https://sate.cirad.fr/S4TE.php). This platform integrates sequence features, signal motifs, and cross-species conservation information from known T4SS effector proteins to assign probability scores for effector potential to genomic products. Based on the scoring results and domain characteristics, candidate T4SS effector proteins with a score greater than 72 were selected for subsequent experimental validation (Supplemental Table S2).

### Translocation of candidate Brucella effectors and their dependence on virB T4SS

**Figure 1.**
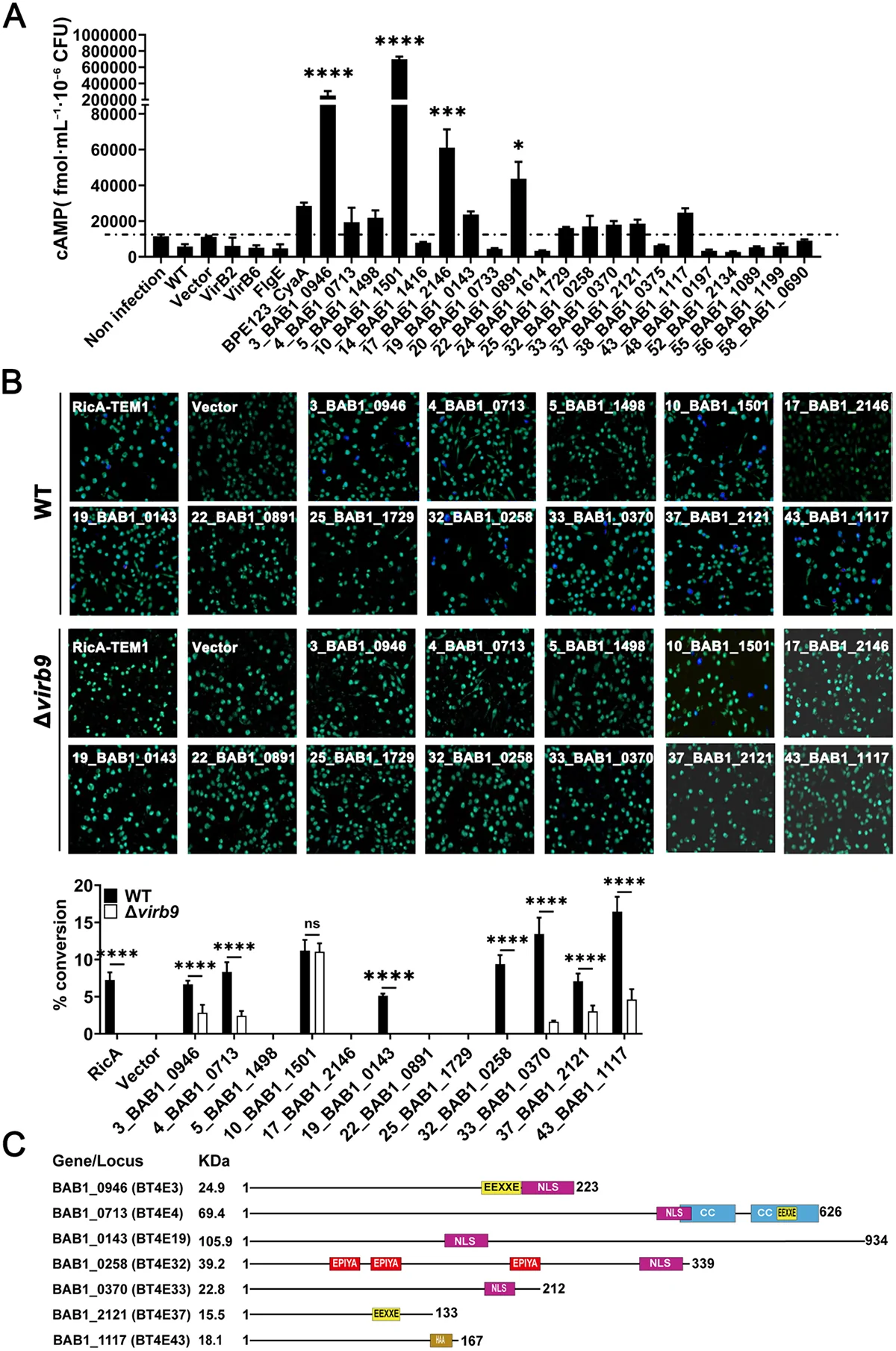
Translocation of *Brucella* putative T4SS effector proteins into RAW264.7 cells.

To validate the translocation of predicted *Brucella* T4SS effectors, we utilized the CyaA reporter system. The assay conditions were validated using the known effector BPE123-CyaA (positive control) and non-secreted proteins VirB2, VirB6, and FlgE (negative controls) [22]. Cyclic AMP (cAMP) levels were measured in RAW264.7 cells infected for 6 h with either wild-type or Δ*virB9* strains expressing the CyaA fusions. All cAMP values were normalized against the intracellular CFU counts of the isogenic WT and Δ*virB9* strains, which were virtually indistinguishable at 6 h post-infection, consistently maintaining around 10^6^ CFU (Supplemental Figure S1C). Infection with *Brucella* strains expressing CyaA fusions of twelve candidates (BAB1_0946, BAB1_0713, BAB1_1498, BAB1_1501, BAB1_2146, BAB1_0143, BAB1_0891, BAB1_1729, BAB1_0258, BAB1_0370, BAB1_2121, and BAB1_1117) resulted in elevated intracellular cAMP levels compared to vector controls (Figure 1(A)), confirming their translocation into the host cytosol.

(A) CyaA fusions to the indicated*Brucella*proteins translocation into RAW264.7 cells.*B. abortus strain 2308*expressing the CyaA fusions to the indicated*Brucella*proteins were used to infect RAW264.7 cells, and the cAMP level of infected cells was determined. A previously identified*virB*effector BPE123 was used as a positive control. Negative Controls included strains expressing VirB2-CyaA, VirB6-CyaA, or FlgE-CyaA hybrid proteins as well as a WT strain containing no plasmid (WT) or a strain containing the CyaA domain alone. Total cAMP levels resulting from translocation of protein fusions were quantified and are expressed as fmol·mL^−1^·10^−6^CFU. (*N* = 3, mean ± SD, one-way ANOVA). *,*p* < 0.05; **,*p* < 0.01; ***,*p* < 0.001; ****,*p* < 0.0001. (B) Representative fluorescence micrographs and quantitative analysis of β-lactamase translocation assays. RAW264.7 cells were infected with B. abortus strain 2308 or the Δ*VirB9*mutant expressing TEM1 fusion proteins to the indicated*Brucella*effectors. The cytosolic translocation of β-lactamase was assessed by fluorescence microscopy, with TEM1-FLAG serving as a negative control and TEM1-RicA as a positive control. Representative fluorescence images from individual assay wells are shown, and the percentage of blue/green fluorescent cells was quantified 16 h p.i. (*N* = 3, mean ± SD, one-way ANOVA). *,*p* < 0.05; **,*p* < 0.01; ***,*p* < 0.001; ****,*p* < 0.0001. (C) Schematic representation of Brucella T4SS Effectors (BT4Es), indicating the corresponding locus, protein lengths, and predicted domains or motifs. CC, coiled coil; EEXXE, E-block; NLS, nuclear signal; EPIYA domain, the Glu-Pro-Ile-Tyr-Ala (EPIYA) motif; HAA, Hydrophobic Amino Acid.

To determine VirB-dependency, we employed the TEM1 β-lactamase reporter assay in WT and T4SS-deficient Δ*Virb9* backgrounds. RAW264.7 cells were infected with TEM1 fusion-expressing wild type *Brucella* strains at an MOI of 1000 for 16 h and processed for fluorescence microscopy analysis [8]. Consistent with the positive control RicA-TEM1, 8 of the 12 candidates exhibited significant substrate conversion in WT-infected cells (Figure 1(B)). Translocation of BAB1_0946, BAB1_0713, BAB1_0143, BAB1_0258, BAB1_0370, BAB1_2121, and BAB1_1117 was significantly abolished in the Δ*Virb9* mutant, confirming them as VirB-dependent effectors. In contrast, BAB1_1501 translocation occurred independently of the VirB system. Four candidates (BAB1_1498, BAB1_2146, BAB1_0891, and BAB1_1729) showed translocation in the CyaA assay but not in the TEM1 assay, suggesting tag-specific interference. Expression of fusion proteins was verified by Western blot (Supplemental Figure S1A-B).

Based on S4TE prediction ranks, we named these *B**rucella* T4SS Effectors (BT4E) BT4E3 (BAB1_0946), BT4E4 (BAB1_0713), BT4E19 (BAB1_0143), BT4E32 (BAB1_0258), BT4E33 (BAB1_0370), BT4E37 (BAB1_2121), and BT4E43 (BAB1_1117). Domain analysis revealed diverse structural features, including E-blocks (BT4E3, BT4E4, BT4E37), nuclear localization signals (BT4E3, BT4E4, BT4E19, BT4E32, BT4E33), coiled-coil domains (BT4E4), EPIYA motifs (BT4E32), and C-terminal HAA regions (BT4E37, BT4E43) (Figure 1(C). Sequence alignment indicated high conservation (>99.6% identity) for all effectors among classical *Brucella* species, with the exception of BT4E32, which is detectable in *B. abortus* 2308, A19, and *B. ovis* ATCC 25840 and shares 99.8%-100% identity among these three *Brucella* species but is absent in the *B. suis* S2 strain, *B. melitensis* 2007BM, *Brucella* canis strain GB1, *Brucella* microti CCM 4915 and *Brucella* pinnipedialis strain 23a-1 (Supplemental Table S3).

### BT4E19 is essential for Brucella growth and cell morphology in vitro

To investigate the roles of the newly identified effectors in *Brucella* growth and cell morphology, we generated in-frame deletion mutants (Δ*bt4e*) along with their corresponding complemented strains (Δ*bt4e::bt4e*). Deletion mutants for BT4E4, BT4E19, BT4E32, BT4E33, and BT4E43 were successfully generated; however, attempts to generate deletions of BT4E3 and BT4E37 were unsuccessful. As demonstrated by Sternon et al., these two genes are essential in *Brucella*, which explains why they could not be deleted [25].

**Figure 2.**
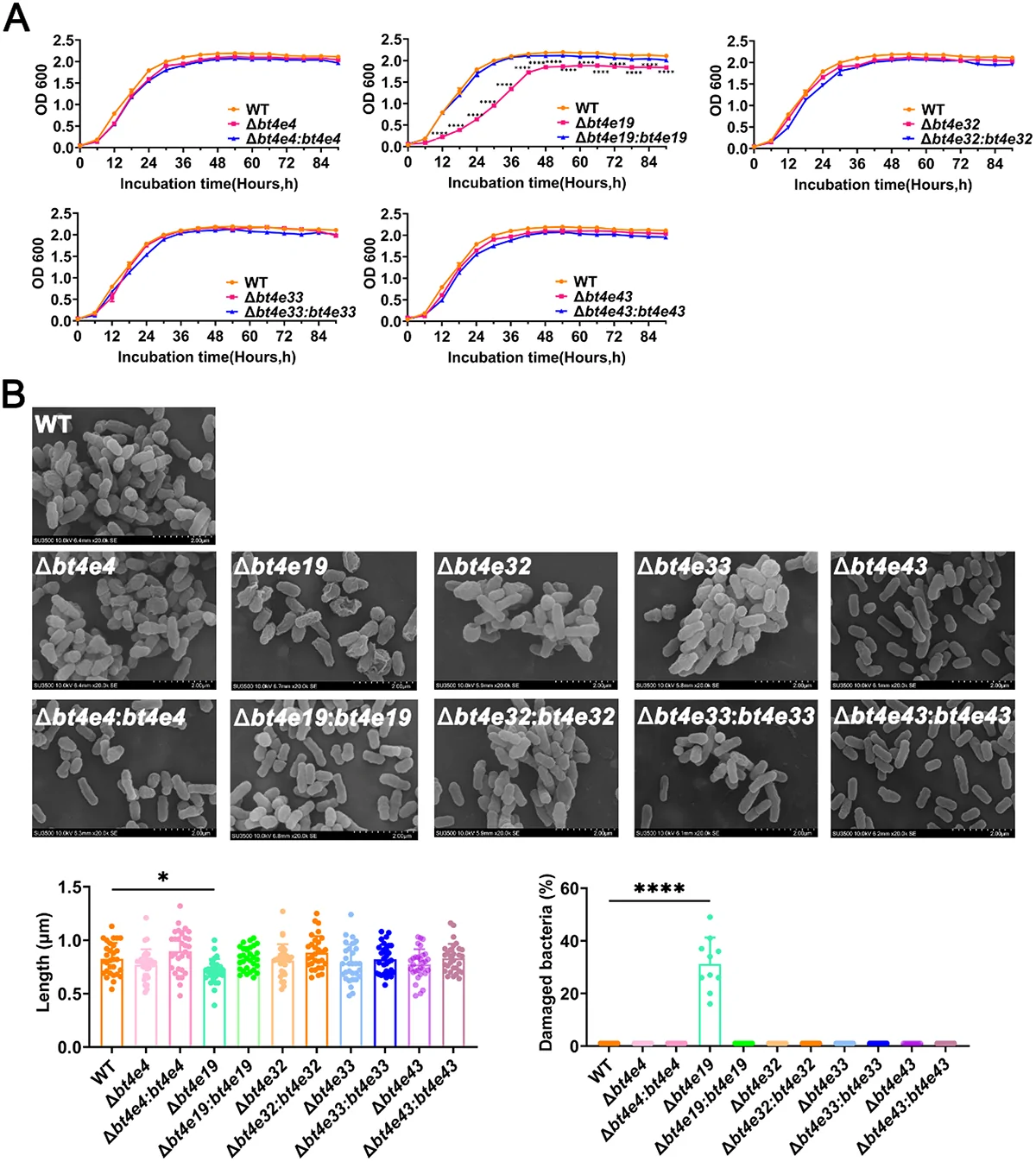
BT4E effectors affect Brucella replication and bacterial morphology in vitro.

Growth kinetics in TSB revealed that deletion of *bt4e19* significantly impaired replication, a defect restored in the complementation strain (Figure 2(A)), which is consistent with the essentiality of BT4E19 (BAB1_0143) in Sternon’s study [25]. Scanning electron microscopy (SEM) showed that the Δ*bt4e19* mutant exhibited severe morphological abnormalities, including membrane swelling, rupture, and reduced cell length (mean length: 0.709 μm vs 0.825 μm for WT; *N* = 30). The length of Δ*bt4e19:bt4e19* was recovered to 0.827 μm suggesting that the decreased OD_600_ value of Δ*bt4e19* may be caused by changed bacterial size and shape, and the deletion of *bt4e19* altered the phenotype and growth (Figure 2(B)). In contrast, the Δ*bt4e4*, Δ*bt4e32*, Δ*bt4e33*, and Δ*bt4e43* mutants displayed growth rates and cell morphologies indistinguishable from the WT strain. These results suggest that BT4E19 is critical for maintaining normal bacterial growth and structural integrity, whereas the other effectors are dispensable for these processes in vitro.

(A) Each volume of*Bacterial*cultures was taken at 6 h interval to determine the absorbance value at 600 nm in TSB. The bacterial OD_600_values were statistically analyzed (*N* = 3, mean ± SD, one-way ANOVA). (B) Representative scanning electron microscopy (SEM) images and quantitative analysis of bacterial morphology. SEM images of the WT strain, the Δ*bt4e*mutant, and the complemented strain (Δ*bt4e:bt4e*) are shown. Bacterial length and the proportion of swollen or damaged bacteria were measured and statistically analyzed (*N* = 30, mean ± SD, one-way ANOVA). *,*p* < 0.05; **,*p* < 0.01; ***,*p* < 0.001; ****,*p* < 0.0001; ns, non-significant difference.

### Deletion of bt4e19 or bt4e43 compromises Brucella lipopolysaccharide (LPS) synthesis

**Figure 3.**
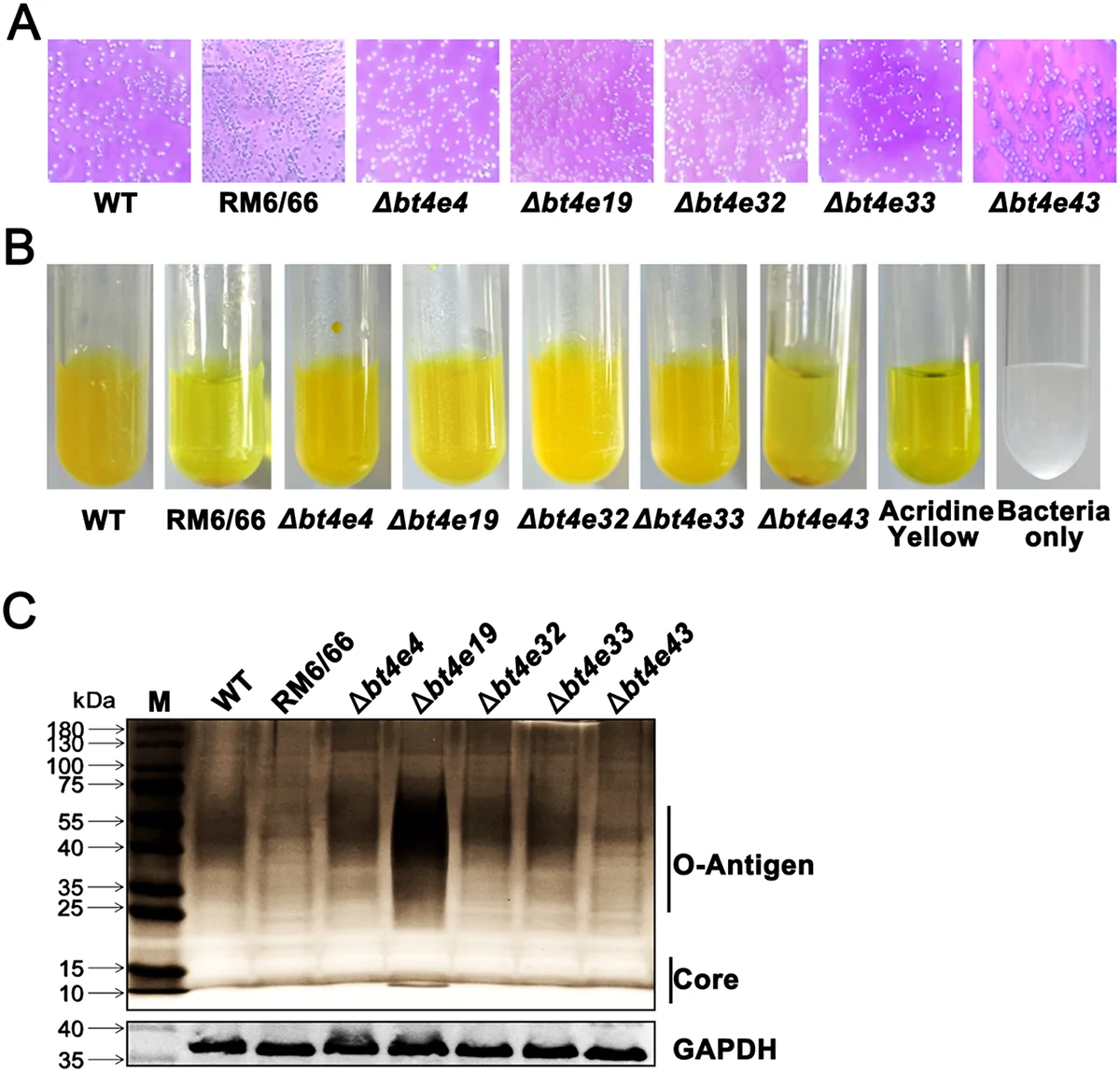
Analysis of lipopolysaccharide (LPS) integrity in WT and effector deficient Brucella strains.

LPS is a major outer-membrane component of Gram-negative bacteria and a critical *Brucella* virulence determinant [26]. To assess the impact of these effectors on LPS integrity, crystal violet staining was performed. On a light purple background, smooth-type colonies with intact LPS displayed a translucent light milky white appearance, whereas rough-type colonies lacking the O-antigen exhibited blue to purple staining. Among the mutants tested, Δ*bt4e43* showed blue staining indistinguishable from that of the rough reference strain RM6/66. In contrast, the Δ*bt4e4*, Δ*bt4e19*, Δ*bt4e32*, and Δ*bt4e33* mutants appeared unstained, with neat, rounded colony edges and a milky white hue characteristic of smooth-type *Brucella* (Figure 3(A)). Consistent with these findings, the acriflavine agglutination assay revealed pronounced agglutination for both RM6/66 and the Δ*bt4e43* mutant, whereas the other mutants did not agglutinate (Figure 3(B)). To further assess the impact of these effectors on LPS biosynthesis, LPS was extracted from the indicated strains and analyzed by silver staining, with *Brucella* GAPDH used as a loading control. Silver-stained LPS profiles demonstrated that the Δ*bt4e43* mutant exhibited substantially reduced O-antigen production relative to the WT strain, displaying an LPS profile consistent with that of the rough strain RM6/66. By contrast, the Δ*bt4e19* mutant displayed a lower apparent molecular weight of the core oligosaccharide and increased abundance of both core oligosaccharide and O-antigen bands (Figure 3(C)). In summary, deletion of *bt4e19* resulted in alterations in core oligosaccharide structure and changes in LPS abundance, whereas deletion of *bt4e43* impaired O-antigen biosynthesis or stability, resulting in an R-like phenotype with compromised LPS integrity.

(A) Bacteria cultured to the logarithmic phase were spread on TSA plates. After single colonies grew, crystal violet staining was performed to observe the staining of single colonies. (B)*Brucella*bacterial pellets were collected, mixed with an equal volume of 1:2000 acriflavine solution, heated at 80°C for 2 h, and then the bacterial precipitation was observed. (C)*Brucella*lipopolysaccharide (LPS) was extracted and subjected to silver staining. The*Brucella*rough strain RM6/66 as a control, Detection of *Brucella* GAPDH was used as a loading control.

### Brucella T4SS effectors exhibit differential roles in environmental stress tolerance

**Figure 4.**
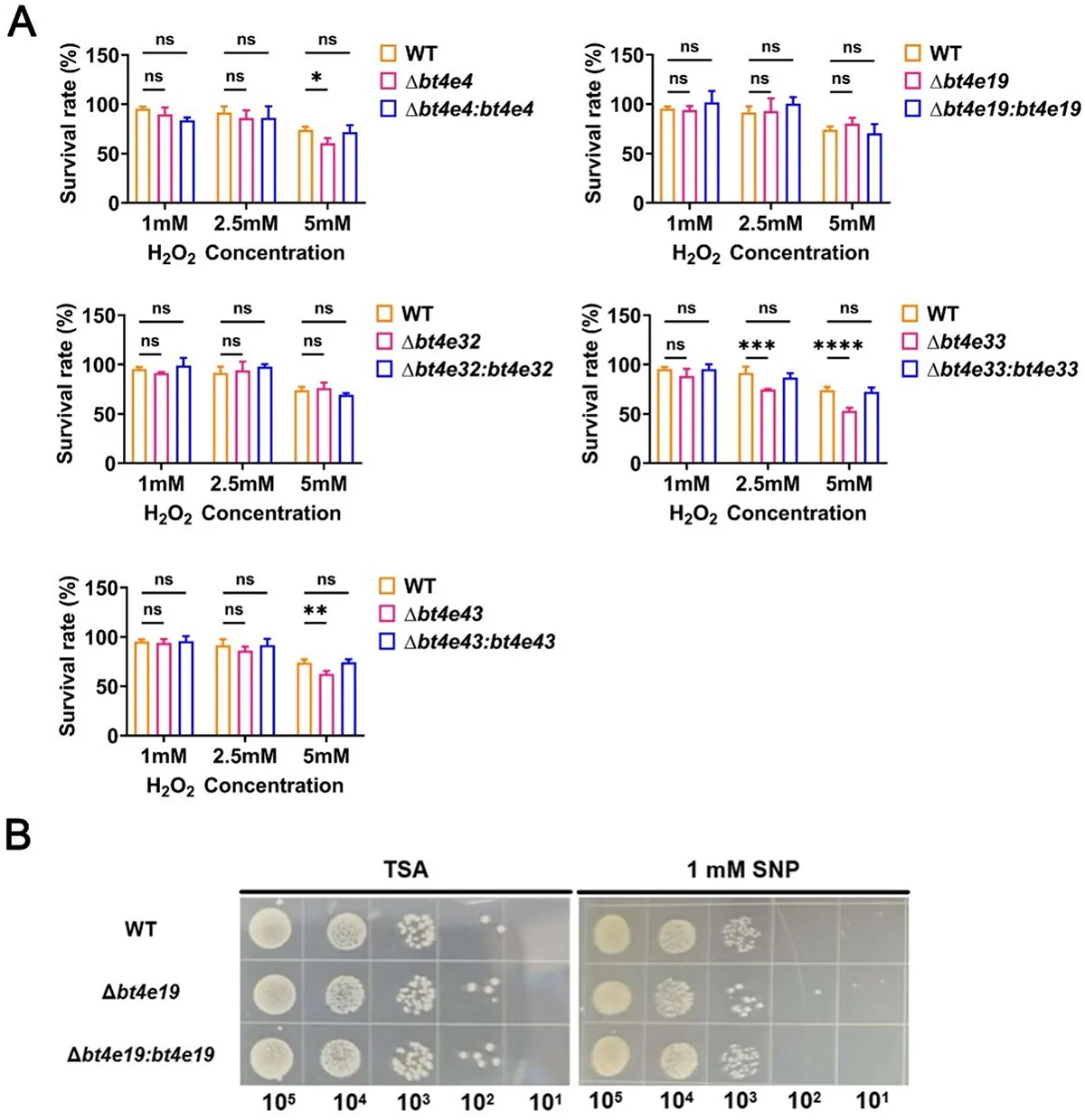
BT4E effectors affect *Brucella* stress tolerance.

As an intracellular pathogen, *Brucella* survival and pathogenesis depend on its capacity to withstand diverse microenvironmental stresses [27]. To evaluate the role of effectors in coping with microenvironmental stresses, we assessed the survival of deletion mutants under various conditions. Upon exposure to oxidative stress (H_2_O_2_), the Δ*bt4e33* mutant exhibited significantly reduced survival at both 2.5 mM and 5 mM H_2_O_2_, whereas the Δ*bt4e4* and Δ*bt4e43* mutant showed a significant reduction at 5 mM, compared to the WT strain. Complementation with the respective wild-type genes restored oxidative stress tolerance to WT levels (Figure 4(A)), while Δ*bt4e19* and Δ*bt4e32* showed no significant sensitivity to H_2_O_2_. Under nitrosative stress conditions induced by 2 mM sodium nitroprusside (SNP), only the Δ*bt4e19* mutant displayed significantly reduced tolerance (Figure 4(B) and Supplemental Figure S2A). No significant differences in survival or growth were observed among the strains when subjected to osmotic stress (100 mM NaCl), membrane destabilization (2 mM EDTA), or acidic environments (pH 4.5/5.5/6.5) (Supplemental Figure S2A-B). These results indicate that BT4E4, BT4E33, and BT4E43 contribute to resistance against oxidative stress, while BT4E19 is specifically required for tolerance to nitrosative stress.

(A) Survival of WT strain, Δbt4e mutant, and Δbt4e complemented strain (Δ*bt4e:bt4e*) under hydrogen peroxide stress. (*N* = 3, mean ± SD, one-way ANOVA). (B) Representative figures of*Brucella*survival on TSA plate containing SNP. *,*p* < 0.05; **,*p* < 0.01; ***,*p* < 0.001; ****,*p* < 0.0001; ns, non-significant difference.

### Deletion of bt4e19 or bt4e43 compromises the intracellular replication capacity of Brucella

**Figure 5.**
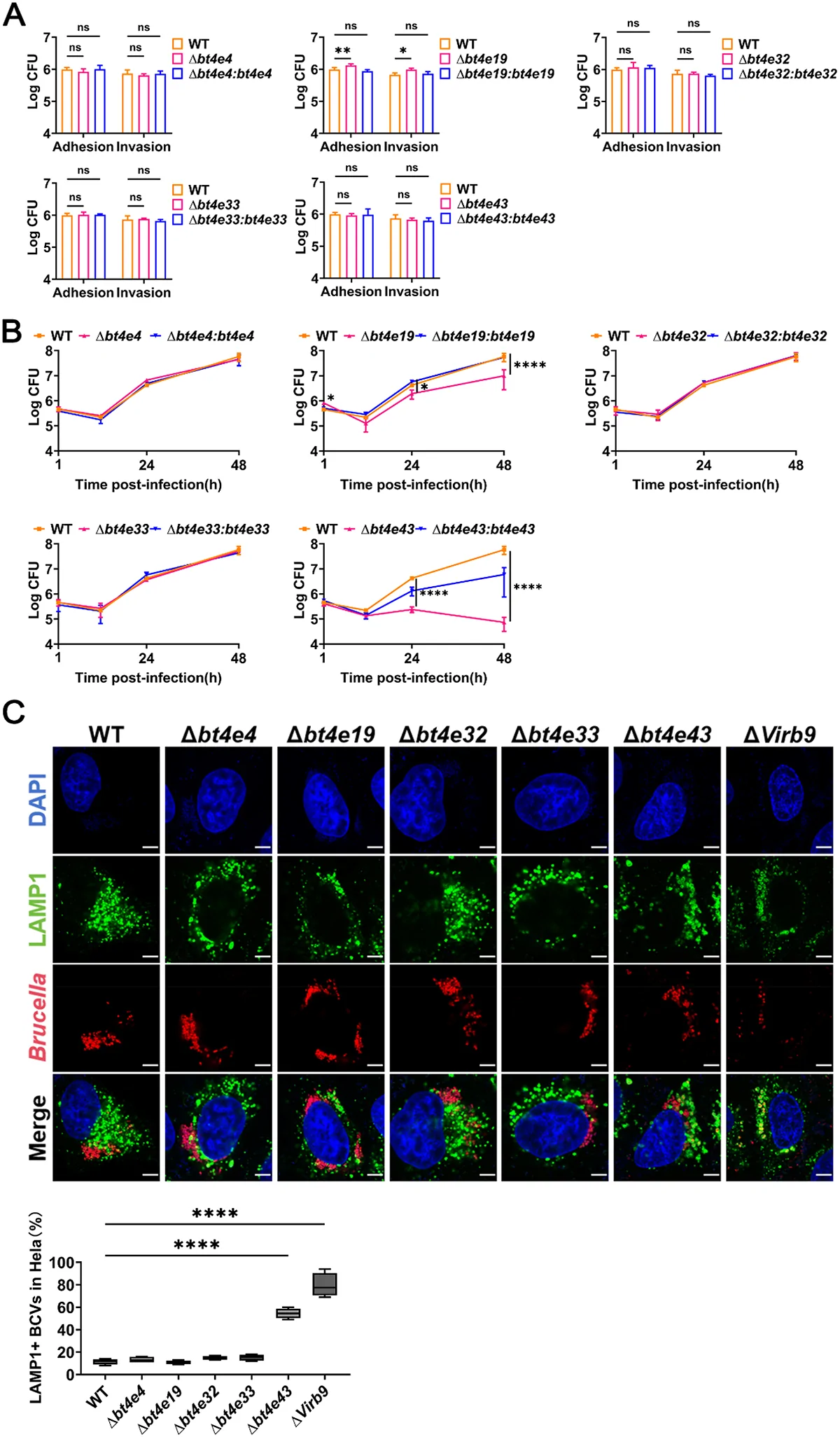
BT4E effectors contribute to Brucella intracellular survival and trafficking.

To delineate the contribution of effectors to infection, we assessed adhesion, invasion, and intracellular replication in macrophages. The Δ*bt4e19* mutant exhibited statistically enhanced adhesion (*p* < 0.01) and invasion (*p* < 0.05) efficiency compared to the WT strain (Figure 5(A)); However, it failed to sustain intracellular replication, resulting in significantly reduced bacterial loads at 48 h p.i (Figure 5(B)). This phenotype was specific, as complementation restored both invasion and replication dynamics to WT levels. The Δ*bt4e43* mutant displayed normal initial entry and early survival but exhibited a significant replication defect at 24 h p.i. (*p* < 0.001) and 48 h p.i. (*p* < 0.0001), and complementation partially restored the defect of intracellular replication at 24 h p.i. and 48 h p.i. No significant differences in adhesion or intracellular growth were observed for Δ*bt4e4*, Δ*bt4e32*, or Δ*bt4e33*.

(A) Ability of WT strain, Δ*bt4e*mutant, and Δ*bt4e*complemented strain (Δ*bt4e:bt4e*) to adhere and invade to Raw264.7 cells (*N* = 3, mean ± SD, two-way ANOVA). (B) WT strain, Δ*bt4e*mutant, and Δ*bt4e*complemented strain (Δ*bt4e:bt4e*) in RAW264.7 cells. Intracellular CFUs were counted at 1 h, 24 h, and 48 h p.i. (*N* = 3, mean ± SD, two-way ANOVA). (C) Co-localization of*Brucella*with LAMP1 in HeLa cells at 24 hpi. About 100 bacteria were counted randomly per coverslip and the percentage of LAMP-1 co-localized*Brucella*-containing vacuoles (BCV) was determined. Assays were performed in triplicate. Representative confocal microscopy images showing co-localization of*Brucella*and lysosome-associated membrane protein (LAMP1). Pearson’s correlation coefficient for BCV-LAMP1 co-localization is shown (*N* = 100, mean ± SD, one-way ANOVA). Scale bar, 10 μm. *,*p* < 0.05; **,*p* < 0.01; ***,*p* < 0.001; ****,*p* < 0.0001; ns, non-significant difference.

Escaping lysosomal degradation is a critical prerequisite and a key step for *Brucella* to establish its intracellular replicative niche [28,29]. To determine if the observed defects resulted from aberrant intracellular trafficking, we analyzed the recruitment of the lysosomal marker LAMP1 to *Brucella*-containing vacuoles (BCVs). Immunofluorescence assays revealed that BCVs containing Δ*bt4e4*, Δ*bt4e19*, Δ*bt4e32*, and Δ*bt4e33* efficiently excluded LAMP1 (<20% co-localization), similar to the WT. In contrast, the Δ*bt4e43* mutant exhibited high frequencies of LAMP1 co-localization (~60%), comparable to the T4SS-deficient Δ*Virb9* strain (Figure 5(C)). These results indicate that the altered bacterial entry and general survival associated with the Δ*bt4e19* mutant likely stem from its generally compromised physiological state and envelope integrity, comparable to the Δ*wadC* mutant that affects the LPS core and thereby attenuates *Brucella* virulence [13]. In contrast, the impaired avoidance of lysosomal trafficking observed in the Δ*bt4e43* mutant is highly consistent with the well-established pleiotropic phenotypes of rough *Brucella* LPS mutants [30].

### The mutants Δbt4e19 and Δbt4e43 exhibit decreased pathogenicity in mice

**Figure 6.**
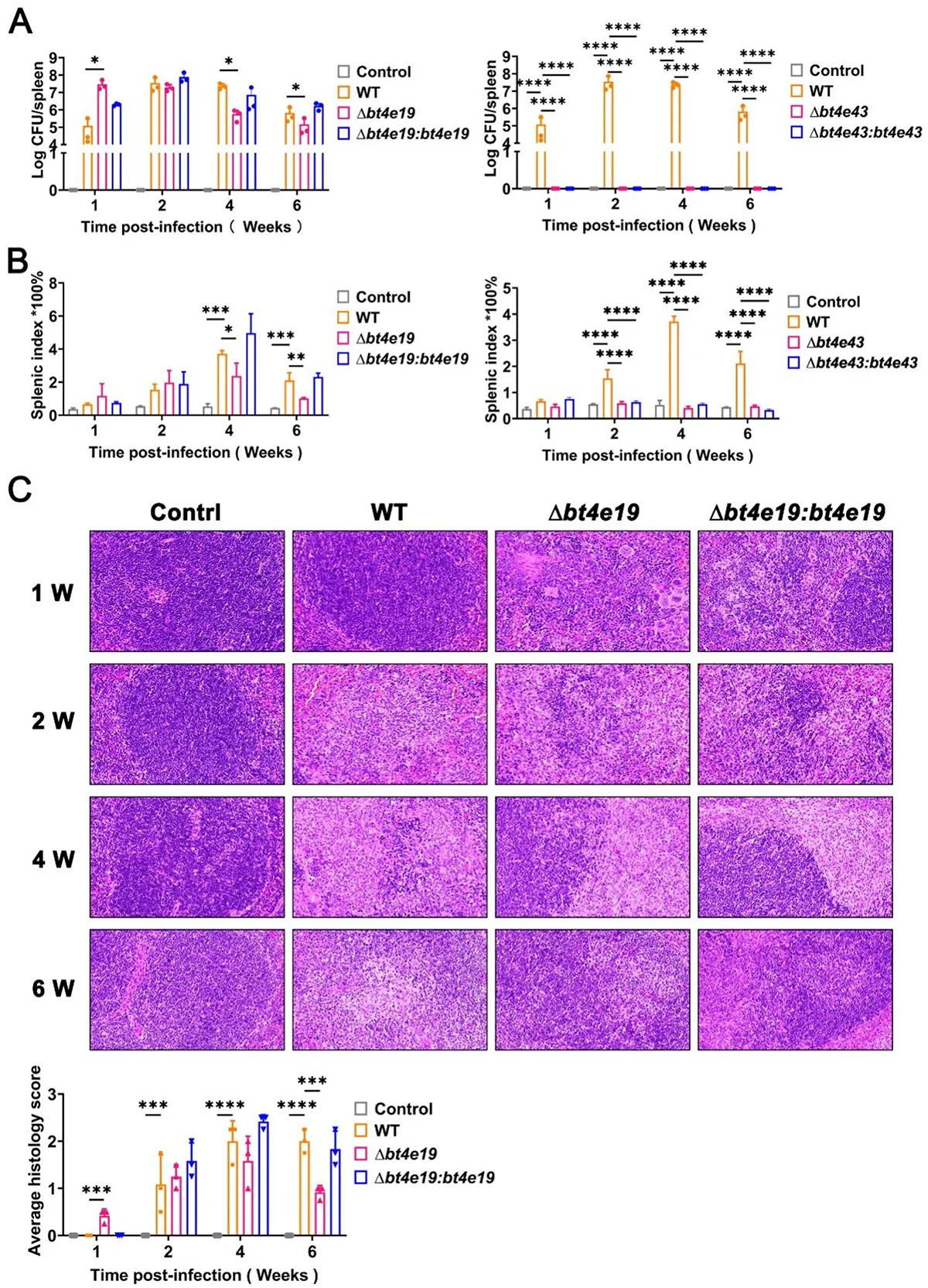
BT4E19 and BT4E43 were involved in Brucella virulence in mice infection models.

Given that BT4E19 and BT4E43 are required for efficient intracellular replication, we next evaluated their contribution to virulence using a mouse infection model. The Δ*bt4e19* mutant exhibited a biphasic infection profile: splenic bacterial loads and weights were significantly higher than WT or complemented strain Δ*bt4e19::bt4e19* at 1-week p.i., consistent with enhanced *in vitro* invasion, but became comparable at 2 weeks p.i., and were significantly reduced during the chronic phase (4 and 6 weeks p.i.) (Figure 6(A and B)). These data indicate that *bt4e19* is essential for the maintenance of chronic infection. In contrast, the Δ*bt4e43* mutant was unable to establish infection. No viable bacteria were recovered from spleens, and no splenomegaly was observed at any time point assessed (1–4 weeks p.i.). Complementation failed to rescue this colonization defect *in vivo*.

(A) The splenic CFUs of WT strain, Δ*bt4e*mutant, and Δ*bt4e*complemented strain (Δ*bt4e:bt4e*) infected mice at 1, 2, 4 and 6 weeks p.i. (B) The spleen weight of WT strain, Δ*bt4e*mutant, and Δ*bt4e*complemented strain (Δ*bt4e:bt4e*) infected mice at 1, 2, 4, and 6 weeks p.i. (C) The spleen of mice infected with WT strain, Δ*bt4e19*mutant, and Δ*bt4e19*complemented strain (Δ*bt4e19:bt4e19*) strain at 1, 2, 4, and 6 weeks post infection were collected. Hematoxylin and eosin staining was used (×10 and ×20 magnification). Sections from spleen were scored for severity of histiocytic inflammation from 1 to 4 after p.i. (*N* = 3, mean ± SD, one-way ANOVA). *,*p* < 0.05; **,*p* < 0.01; ***,*p* < 0.001; ****,*p* < 0.0001; ns, non-significant difference.

Histopathological analysis of WT-infected spleens revealed progressive architectural disruption, lymphocyte depletion, and fibrosis. Consistent with bacterial loads, Δ*bt4e19* induced severe pathology peaking at 1-week p.i. which partially resolved at later time points, whereas the complemented strain induced WT-like lesions (Figure 6(C)). In contrast, mice inoculated with Δ*bt4e43* or its complement displayed no significant pathological changes compared to uninfected controls (Supplemental Figure S3A).

### The mutants Δbt4e4, Δbt4e19, and Δbt4e43 induce inflammatory cell death

**Figure 7.**
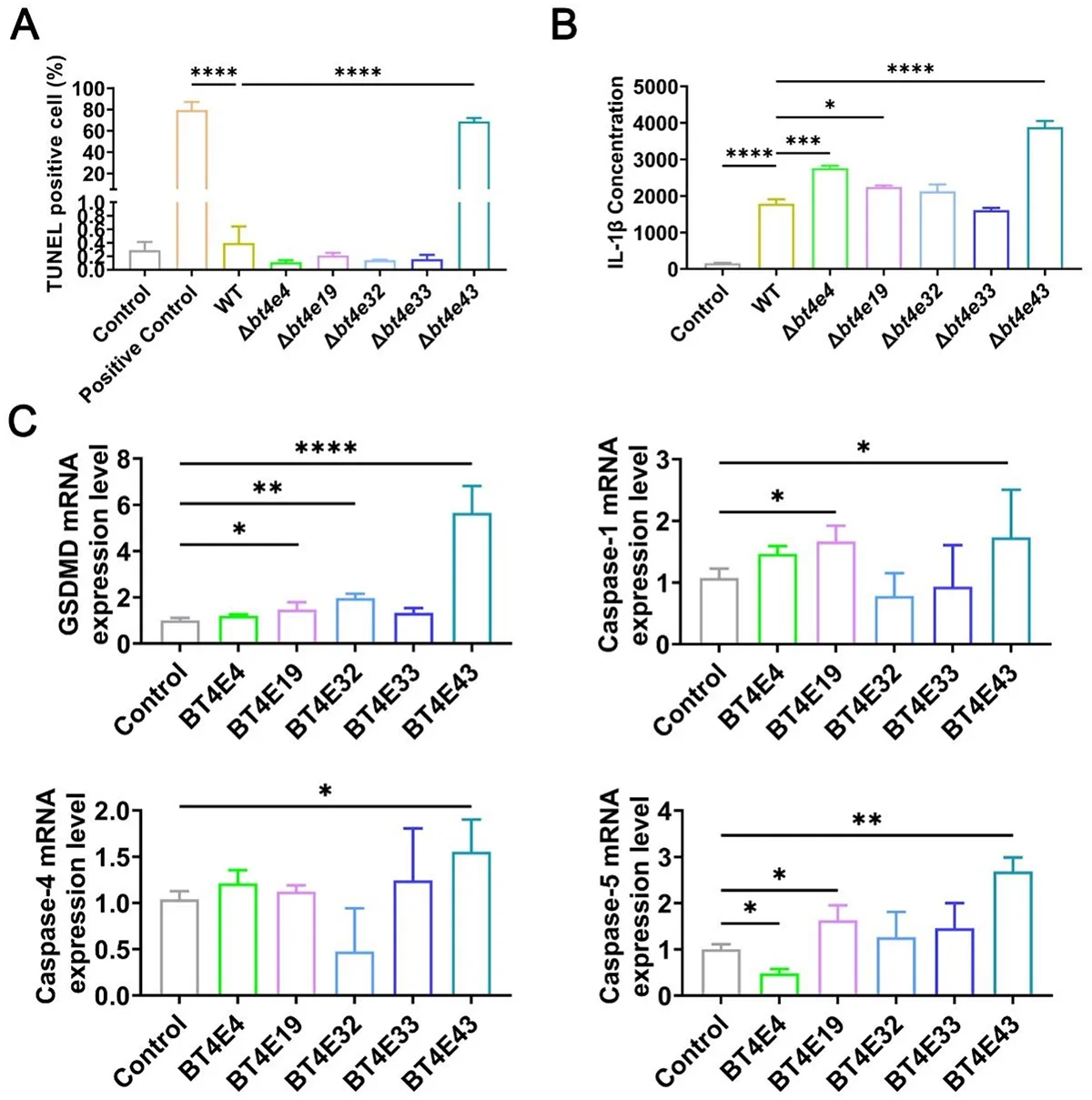
BT4E effectors modulate inflammatory cell death.

For intracellular pathogens, regulating host cell death is critical for dissemination and immune evasion [31,32]. To evaluate the role of BT4E effectors in apoptosis, THP-1 cells were infected with deletion mutants and analyzed by TUNEL assay. The Δ*bt4e43* mutant induced significantly higher levels of apoptosis compared to the wild-type (WT) strain (Figure 7(A)). However, ectopic expression of individual effectors in Expi293F cells did not alter baseline apoptosis levels (Supplemental Figure S4A), suggesting that the pro-apoptotic phenotype of Δ*bt4e43* during infection is likely an indirect consequence of bacterial physiological defects (e.g. compromised LPS) rather than direct modulation of apoptotic pathways [24].

(A) Detection of apoptosis in RAW264.7 cells infected with BT4E effector-deficient strains for 24 hours by flow cytometry. Uninfected cells were used as controls. (B) Expression levels of pyroptosis-related genes in A549 cells 24 h after ectopic expression of BT4E effectors were determined by quantitative PCR (qPCR). Empty vector served as the control. (*N* = 3, mean ± SD, one-way ANOVA). *,*p* < 0.05; **,*p* < 0.01; ***,*p* < 0.001; ****,*p* < 0.0001; ns, non-significant difference.

We next assessed pyroptosis by measuring IL-1β release from infected THP-1 cells. Infection with Δ*bt4e4*, Δ*bt4e19*, or Δ*bt4e43* resulted in significantly elevated IL-1β levels relative to the WT, indicating enhanced pyroptosis (Figure 7(B)). To investigate direct transcriptional modulation, we expressed these effectors in A549 cells and quantified pyroptosis-related mRNA. BT4E4 expression significantly suppressed *Caspase5* transcription, consistent with its potential role in inhibiting pyroptosis. Conversely, BT4E19 upregulated *Caspase1* and *Caspase5*, and BT4E43 enhanced the expression of *GSDMD*, *Caspase1*, *Caspase4*, and *Caspase5* (Figure 7(C)). These findings indicate that BT4E4 is capable of modulating *Caspase5* associated pyroptotic signaling when expressed in host cells. In contrast, the enhanced pyroptosis observed during infection with the Δ*bt4e19* and Δ*bt4e43* mutants likely results from the indirect activation of host immunity, which is more likely an indirect consequence of their compromised cell envelope integrity and altered LPS structures [33–35].

## Discussion

*Brucella spp*. establish persistent intracellular infection by delivering effector proteins into host cells via the VirB type IV secretion system (T4SS) [5], thereby coordinating bacterial physiology with host immune responses. In this study, we combined bioinformatic prediction using S4TE 2.0 with two orthogonal translocation assays (CyaA and TEM1) to identify seven novel T4SS substrates [8], designated BT4E3 (BAB1_0946), BT4E4 (BAB1_0713), BT4E19 (BAB1_0143), BT4E32 (BAB1_0258), BT4E33 (BAB1_0370), BT4E37 (BAB1_2121), and BT4E43 (BAB1_1117). We focused functional analyses on five of these effectors BT4E4, BT4E19, BT4E32, BT4E33, and BT4E43 and found that they modulate both bacterial core processes (including lipopolysaccharide biosynthesis, stress tolerance, and cell morphology) and host cellular pathways (such as inflammatory cytokine production).

The Δ*bt4e19* mutant exhibited pleiotropic defects similar to those observed in *alr* or *wbkC* deletion mutants [24,33], including impaired growth, altered cell morphology, reduced tolerance to nitrosative stress (SNP), and modifications in the LPS core oligosaccharide structure. Plasmid-based complementation restored these phenotypes, confirming that they result from the loss of *bt4e19*. We propose that BT4E19 contributes to outer membrane integrity, potentially through effects on LPS core organization. *In vivo*, Δ*bt4e19* displayed a biphasic infection profile: splenic bacterial loads were higher than WT at 1 week p.i. but significantly reduced by 4–6 weeks. This “early increase, late decline” likely results from LPS alterations that expose surface adhesins, which enhance initial host cell invasion and colonization [33,34], while simultaneously rendering the bacterium sensitive to the ROS/RNS-rich environment of the chronic splenic niche [34]. This trajectory resembles that of rough *Brucella* mutants (e.g. Δ*wbkA*), which are internalized efficiently but fail to sustain chronic infection [36].

In contrast, the Δ*bt4e43* mutant was avirulent, failing to colonize the spleen at any time point. This phenotype, mirroring T4SS-deficient strains (e.g. Δ*Virb9*), underscores the essentiality of BT4E43 for systemic infection [37,38]. *In vitro*, Δ*bt4e43* failed to exclude the lysosomal marker LAMP1 from *Brucella*-containing vacuoles (BCVs). Notably, plasmid-mediated complementation did not restore *in vivo* virulence or LAMP1 exclusion [39]. This failure may stem from polar effects on downstream operon genes, non-physiological overexpression disrupting effector stoichiometry, or the severe early bottleneck caused by O-antigen loss, which renders the mutant susceptible to immediate immune clearance. Therefore, the lack of complementation in vivo does not necessarily preclude a role for BT4E43 during early stages of infection. Future studies employing chromosomal in situ complementation or reconstructing endogenous regulatory elements at the native genomic locus will help disentangle these variables and establish the physiological role of BT4E43 within the infection niche.

Interestingly, while the Δ*bt4e33* mutant exhibited significant sensitivity to exogenous oxidative stress in vitro, this phenotype was not prominently reflected as a severe intracellular survival defect in RAW264.7 macrophages. The high-dose H_2_O_2_ exposure in vitro differs fundamentally from the spatially confined and physiologically regulated ROS burst within the macrophage phagosome; if the mutant successfully establishes its ER-derived replicative niche [40,41], it physically evades the high-ROS degradative compartment. This functional redundancy is well supported by previous findings; for instance, combining deletions in multiple effectors, such as *bspA*, *bspB*, and *bspF* [8], results in additive effects on the restoration of secretion inhibition and intracellular replication defects. Furthermore, the context-dependent nature of these effectors is highlighted by the observation that BspB is dispensable for rBCV biogenesis and bacterial replication in the absence of RicA [42], collectively demonstrating the complex interplay and compensatory mechanisms within the T4SS network.

Regarding host cell death, infection with Δ*bt4e4*, Δ*bt4e19*, or Δ*bt4e43* increased IL-1β release, and Δ*bt4e43* also enhanced apoptosis. However, ectopic expression of these effectors produced distinct results. BT4E4 suppressed *Caspase5* transcription, suggesting that it may play a direct role in inhibiting pyroptosis. However, it remains unclear whether BT4E4 regulates the *Caspase5* promoter directly as a transcription factor or indirectly through upstream signaling pathways. This question warrants further investigation in future studies. Conversely, BT4E19 and BT4E43 upregulated pro-inflammatory genes (*Caspase1*, *Caspase4*, *Caspase5*, *GSDMD*) and failed to induce apoptosis. This discrepancy suggests that the heightened cell death observed during infection with Δ*bt4e19* or Δ*bt4e43* likely reflects indirect immune activation associated with bacterial envelope defects, rather than loss of direct effector‑mediated immune suppression.

Limitation: Although the TEM-1 β-lactamase and CyaA reporter assays currently employed are the most widely accepted approaches for identifying *Brucella* T4SS effectors, the altered subcellular localization or physiological state of the virB mutant may indirectly influence the detection of these effector proteins in host cells. Additionally, while the CyaA assay required a higher MOI for the Δ*Virb9* mutant to overcome intracellular clearance, and all data were rigorously normalized to intracellular CFUs to account for this, we acknowledge that extremely high bacterial loads could theoretically impose physical stress on host cells or alter background signaling. Therefore, future studies may require more direct methods to validate the translocation of T4SS effector proteins, such as applying confocal laser scanning microscopy to directly visualize their translocation [43].

In summary, this study identifies several novel T4SS substrates and characterizes their intrinsic roles in *Brucella* physiology, particularly in LPS biosynthesis and cell envelope maintenance. By elucidating the role of BT4E19 in envelope integrity and demonstrating that BT4E43 likely influences BCV maturation indirectly via its involvement in LPS biosynthesis, our findings demonstrate that the intracellular phenotypes associated with their deletion are predominantly indirect consequences of compromised envelope integrity and altered bacterial fitness. These results advance the mechanistic understanding of *Brucella* pathogenesis by highlighting the critical importance of LPS structure in balancing bacterial fitness with host immune evasion.

## Supplementary Material

supplemental table S3.xlsx

Supplemental Figure Clean V2.docx

supplemental table S2.xlsx

supplemental table S1.xlsx

Author Checklist.pdf

## Data Availability

All data for this article can be accessed online at https://doi.org/10.6084/m9.figshare.31046704 [38].
