# Supplementary material for: Brucella deploys multiple type IV effectors that simultaneously control bacterial survival and host inflammatory cell death
Source: Virulence. 2026 Aug 28;17(1):2721826. doi: 10.1080/21505594.2026.2721826 (PMC13531981; doi:10.1080/21505594.2026.2721826)
Supplement: Supplementary file 1 [file KVIR_A_2721826_SM4736.pdf]

NOTE: Please save this file locally before filling in the table, DO NOT work on the file within your internet browser as changes will not be saved. Adobe Acrobat Reader (available free here) is recommended for completion.

ARRIVE

The ARRIVE guidelines 2.0: author checklist

The ARRIVE Essential 10

These items are the basic minimum to include in a manuscript. Without this information, readers and reviewers cannot assess the reliability of the findings.

Item		Recommendation	Section/line number, or reason for not reporting
**Study design**	1	For each experiment, provide brief details of study design including:	
		a. The groups being compared, including control groups. If no control group has been used, the rationale should be stated.	Materials and Methods: "Bacterial strains…", "Mice infection", "Mammalian cell culture and bacterial infections"<br>• Groups: WT, Δbt4e mutants, complemented strains, uninfected/PBS controls, RM6/66 (LPS), empty vector/CyaA/TEM1 controls (Fig 1 legend
		b. The experimental unit (e.g. a single animal, litter, or cage of animals).	Experimental unit: single mouse (Mice infection section); single well of cells or single bacterial culture
**Sample size**	2	a. Specify the exact number of experimental units allocated to each group, and the total number in each experiment. Also indicate the total number of animals used.	Mice infection: "Groups of five mice…" → n=5/group/time point; total =20 mice/strain (4 time points)<br>In vitro: "All assays were performed in triplicate" + "biological triplicates"
		b. Explain how the sample size was decided. Provide details of any *a priori* sample size calculation, if done.	Sample size rationale: Based on established murine brucellosis models (consistent with field standards) — implied in Methods and Fig 6 legend
**Inclusion and exclusion criteria**	3	a. Describe any criteria used for including and excluding animals (or experimental units) during the experiment, and data points during the analysis. Specify if these criteria were established *a priori*. If no criteria were set, state this explicitly.	No animals or data points were excluded.
		b. For each experimental group, report any animals, experimental units or data points not included in the analysis and explain why. If there were no exclusions, state so.	All mice that survived to scheduled endpoints were included.
		c. For each analysis, report the exact value of *n* in each experimental group.	• Confirmed in Results: "Data are presented as mean ± SD… (N=5)" (Fig 6A)<br>• In vitro: N=3 (biological replicates) consistently reported (Figs 2–5, Suppl Figs)
**Randomisation**	4	a. State whether randomisation was used to allocate experimental units to control and treatment groups. If done, provide the method used to generate the randomisation sequence.	Mice infection: mice were "randomly assigned" to groups
		b. Describe the strategy used to minimise potential confounders such as the order of treatments and measurements, or animal/cage location. If confounders were not controlled, state this explicitly.	Mice infection section: All mice were age-, sex-, source-matched (BALB/c, female, 4–6 wks, SPF); infections processed in parallel; housed under identical conditions
**Blinding**	5	Describe who was aware of the group allocation at the different stages of the experiment (during the allocation, the conduct of the experiment, the outcome assessment, and the data analysis).	Animal experimental technicians were aware of reason for mouse acquisition from donor animals, but groups not known to those conducting mouse bioassays.
**Outcome measures**	6	a. Clearly define all outcome measures assessed (e.g. cell death, molecular markers, or behavioural changes).	Defined outcomes: Splenic CFU, spleen weight, histopathology score, LPS integrity (crystal violet/silver stain), stress survival (%), intracellular CFU, LAMP1 co-localization, cytokine levels (IL-1β
		b. For hypothesis-testing studies, specify the primary outcome measure, i.e. the outcome measure that was used to determine the sample size.	Not applicable
**Statistical methods**	7	a. Provide details of the statistical methods used for each analysis, including software used.	Statistical Analysis section: "GraphPad Prism 9.0"; one-/two-way ANOVA with Tukey's post-hoc; significance: $p < 0.05$
		b. Describe any methods used to assess whether the data met the assumptions of the statistical approach, and what was done if the assumptions were not met.	Materials and Methods: Statistical analyses
**Experimental animals**	8	a. Provide species-appropriate details of the animals used, including species, strain and substrain, sex, age or developmental stage, and, if relevant, weight.	Mice infection section: "Female BALB/c mice (4–6 weeks old, SPF grade)"
		b. Provide further relevant information on the provenance of animals, health/immune status, genetic modification status, genotype, and any previous procedures.	Mice infection section
**Experimental procedures**	9	For each experimental group, including controls, describe the procedures in enough detail to allow others to replicate them, including:	Materials and Methods desctibedfully details
		a. What was done, how it was done and what was used.	Materials and Methods: i.p. infection with 10^5 CFU; gentamicin protection assay; LPS extraction; CyaA/TEM1 translocation
		b. When and how often.	Materials and Methods: Euthanasia at 1/2/4/6 wks p.i.; CFU at 1/24/48 h p.i.
		c. Where (including detail of any acclimatisation periods).	
		d. Why (provide rationale for procedures).	To assess chronic infection, immune evasion, effector function (stated in Abstract/Results)
**Results**	10	For each experiment conducted, including independent replications, report:	
		a. Summary/descriptive statistics for each experimental group, with a measure of variability where applicable (e.g. mean and SD, or median and range).	All figures: Mean ± SD reported with exact N (e.g., Fig 6A: "N=5, mean ± SD")
		b. If applicable, the effect size with a confidence interval.	Results and Related figure legends

The Recommended Set

These items complement the Essential 10 and add important context to the study. Reporting the items in both sets represents best practice.

Item		Recommendation	Section/line number, or reason for not reporting
**Abstract**	11	Provide an accurate summary of the research objectives, animal species, strain and sex, key methods, principal findings, and study conclusions.	Abstract :Includes species (mouse), strain (BAI
**Background**	12	a. Include sufficient scientific background to understand the rationale and context for the study, and explain the experimental approach.<br>b. Explain how the animal species and model used address the scientific objectives and, where appropriate, the relevance to human biology.	Introduction (implied by context and citations [1–5])<br>Explains T4SS as central virulence determinant; identifies knowledge gap in effector repertoire; justifies use of murine model for chronic brucellosis (clinically relevant)
**Objectives**	13	Clearly describe the research question, research objectives and, where appropriate, specific hypotheses being tested.	Abstract, Results introduction: "To identify nove
**Ethical statement**	14	Provide the name of the ethical review committee or equivalent that has approved the use of animals in this study, and any relevant licence or protocol numbers (if applicable). If ethical approval was not sought or granted, provide a justification.	Ethics statement section:Approved by the Institutional Animal Care and Use Committee at Institute of Animal Science, Chinese Academy of Agricultural Sciences Permission NO: IAS2025-38
**Housing and husbandry**	15	Provide details of housing and husbandry conditions, including any environmental enrichment.	Mice infection section:Mice housed under stan(
**Animal care and monitoring**	16	a. Describe any interventions or steps taken in the experimental protocols to reduce pain, suffering and distress.<br>b. Report any expected or unexpected adverse events.<br>c. Describe the humane endpoints established for the study, the signs that were monitored and the frequency of monitoring. If the study did not have humane endpoints, state this.	Euthanasia by cervical dislocation under anesthesia (sodium pentobarbital)<br>No adverse events reported<br>Humane endpoints: Scheduled euthanasia at predefined time points (1–6 wks); daily health monitoring implied
**Interpretation/ scientific implications**	17	a. Interpret the results, taking into account the study objectives and hypotheses, current theory and other relevant studies in the literature.<br>b. Comment on the study limitations including potential sources of bias, limitations of the animal model, and imprecision associated with the results.	Discusses dual roles of effectors in bacterial fitness and immune evasion<br>Notes limitation: plasmid-based complementation may not fully rescue in vivo phenotype (e.g., Δbt4e43)
**Generalisability/ translation**	18	Comment on whether, and how, the findings of this study are likely to generalise to other species or experimental conditions, including any relevance to human biology (where appropriate).	Abstract, Discussion: Suggests potential for anti-virulence therapy against human/animal brucellosis
**Protocol registration**	19	Provide a statement indicating whether a protocol (including the research question, key design features, and analysis plan) was prepared before the study, and if and where this protocol was registered.	Not registered.A study protocol outlining the research question, key experimental design elements, and analysis plan was developed prior to the initiation of the study; however, it was not prospectively registered in a public repository.
**Data access**	20	Provide a statement describing if and where study data are available.	Data availability statement::Proteomics Proteor
**Declaration of interests**	21	a. Declare any potential conflicts of interest, including financial and non-financial. If none exist, this should be stated.<br>b. List all funding sources (including grant identifier) and the role of the funder(s) in the design, analysis and reporting of the study.	The authors declare that they have no known competing financial interests or personal relationships that could have appeared to influence the work reported in this paper.<br>Funding: CAAS Innovation Project (CAAS-CSLPDCP-202403), China Postdoctoral Science Foundation (2025T180827, GZB20250698)
