# Supplementary material for: Brucella deploys multiple type IV effectors that simultaneously control bacterial survival and host inflammatory cell death
Source: Virulence. 2026 Aug 28;17(1):2721826. doi: 10.1080/21505594.2026.2721826 (PMC13531981; doi:10.1080/21505594.2026.2721826)
Supplement: Supplementary file 2 [file KVIR_A_2721826_SM4739.docx]

**Supplemental Figure 1. Western blot analysis of the expression of TEM1/CyaA-tagged proteins.**

(A) Western blot analysis showing expression of CyaA fusions. Bacterial lysates from *B. abortus* 2308 strain expressing CyaA fusion proteins were resolved using SDS-PAGE and subjected to Western blot analysis with anti-FLAG antibody. GAPDH was used as a loading control. (B) Western blot analysis showing expression of TEM1 fusions. Bacterial lysates from *B. abortus* 2308 and Δ*Virb9* strain expressing TEM1 fusion proteins were resolved using SDS-PAGE and subjected to Western blot analysis with anti-FLAG antibody. GAPDH was used as a loading control. (C) Cells were infected with either wild-type 2308 or the Δ*Virb*9 mutant strains at an MOI of 1000 and 5000, respectively, and intracellular CFUs enumerated at various time points.

**Supplemental Figure 2. Impact of effector deletion affect *Brucella* sensitivity to bactericidal media.**

(A) Representative figures of *Brucella* survival on TSA plate containing different concentrations of EDTA, NaCl, and SNP. (B) Survival of WT strain, Δ*bt4e* mutant, and Δ*bt4e* complemented strain (Δ*bt4e*:*bt4e*) in acidic medium. (N = 3, mean ± SD, one-way ANOVA). ns = non-significant difference.)

**Supplemental Figure 3. BT4E43 was involved in *Brucella* virulence in mice infection models.**

(A) The spleen of mice infected with WT strain, Δ*bt4e43* mutant, and Δ*bt4e43* complemented strain (Δ*bt4e43*:*bt4e43*) strain at 1 and 2 weeks post infection were collected. Hematoxylin and eosin staining was used (×10 and ×20 magnification). Sections from spleen were scored for severity of histiocytic inflammation from 1 to 4 after p.i.. (N=3, mean ± SD, one-way ANOVA). *, *p*<0.05; **, *p*<0.01; ***, *p*<0.001; ****, *p*<0.0001; ns, non-significant difference.

**Supplemental Figure 4. BT4E effectors modulate inflammatory cell death.**

(A) Apoptosis of Expi293F cells 24 h after ectopic expression of BT4E effectors was measured by flow cytometry. Cells transfected with empty vector served as the control. Statistical significance was analyzed by independent samples t-test. *, *p* < 0.05; **, *p* < 0.01; ***, *p* < 0.001; ****, *p* < 0.0001; ns, not significant.
